# Supplementary material for: Top-down descending facilitation of spinal sensory excitatory transmission from the anterior cingulate cortex
Source: Nat Commun. 2018 May 14;9:1886. doi: 10.1038/s41467-018-04309-2 (PMC5951839; doi:10.1038/s41467-018-04309-2)
Supplement: Supplementary file 1 — Supplementary Information [file 41467_2018_4309_MOESM1_ESM.pdf]

Title: Top-down descending facilitation of spinal sensory excitatory transmission from the anterior cingulate cortex

Chen et al.

.

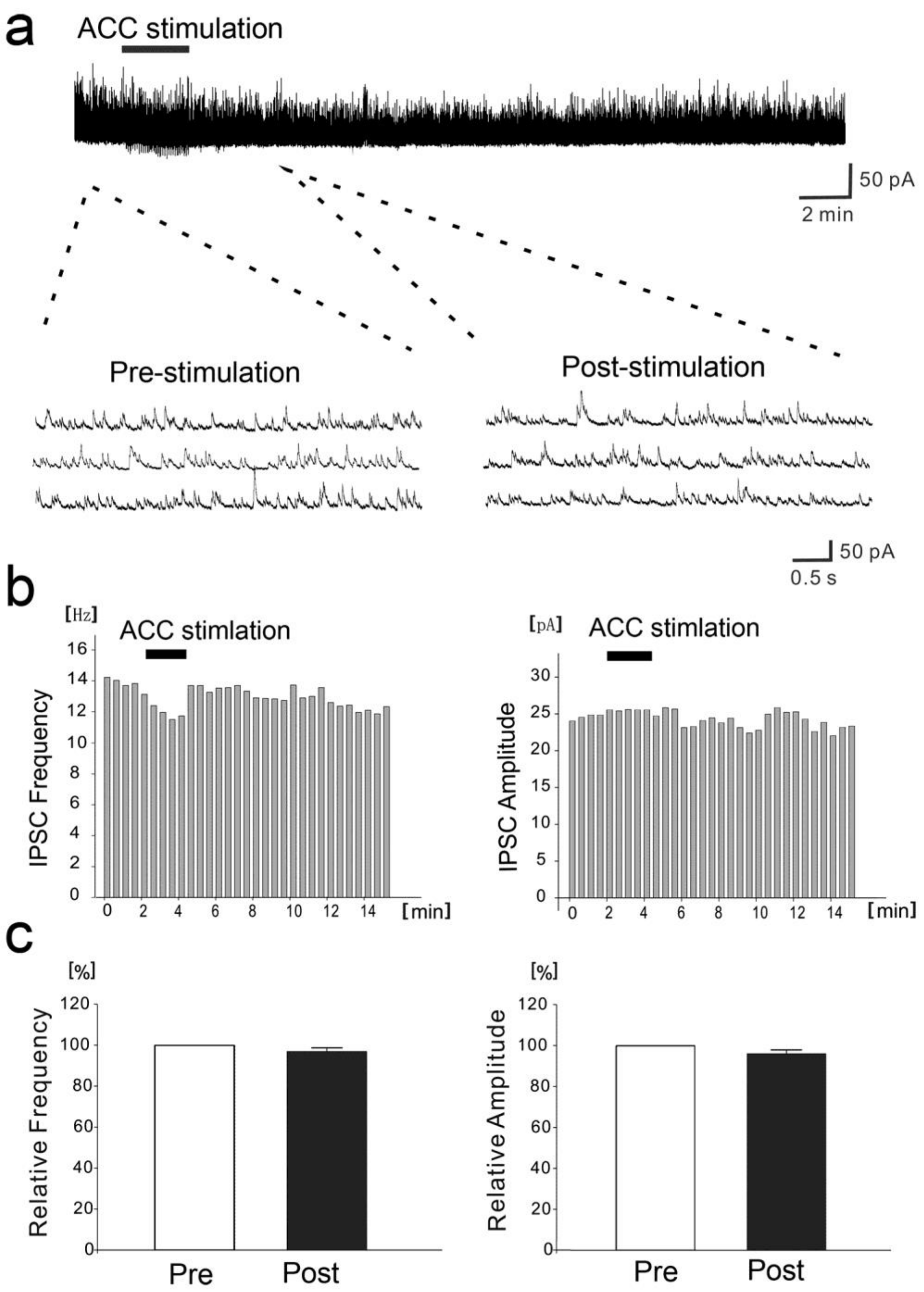

Supplementary Figure 1 | ACC stimulation did not affect sIPSCs on the SDH neurons. (a) One *in vivo* patch clamp recording sample showing that ACC electrical stimulation did not affect the frequency and amplitude of the sIPSCs of SDH neurons in naïve rats. **(b)** Histogram figures showing the frequency and amplitude of the sample sIPSCs in **(a)** before and after ACC stimulation. **(c)** Summarized results from 7 SDH neurons.

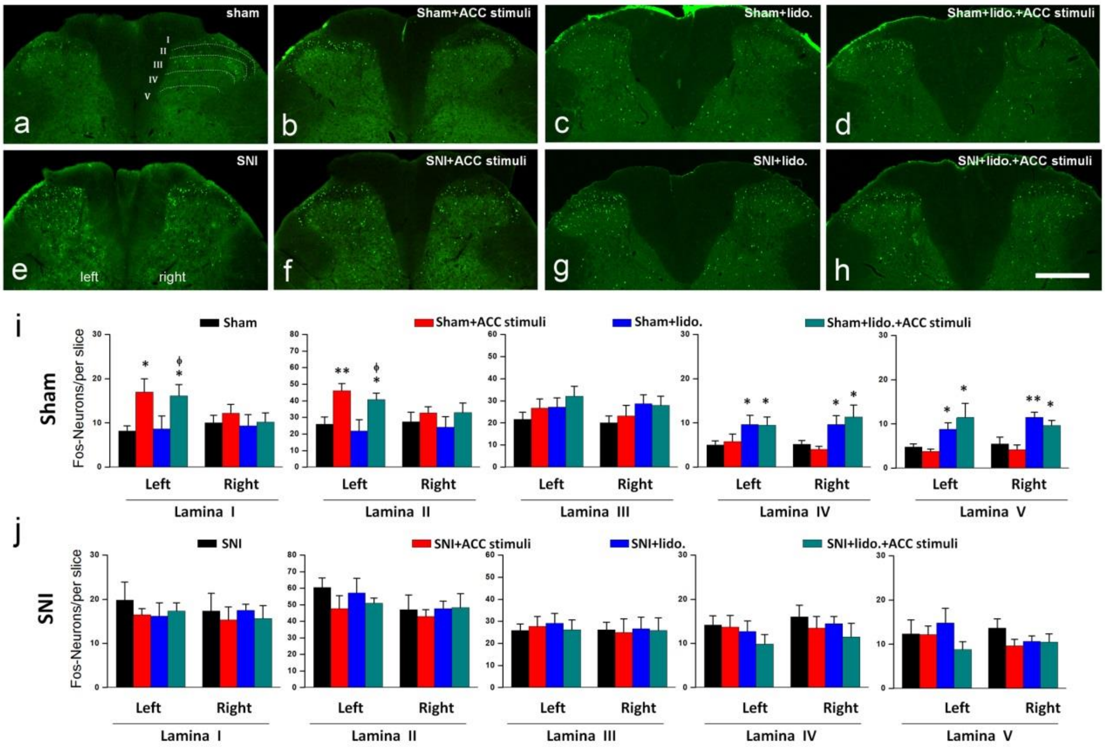

Supplementary Figure 2 | The Fos expression on the spinal dorsal horn after ACC stimulation and RVM blockade. (a-d) Sample photos showing the Fos expression in the L5 SDH of sham operated rats. **(e-h)** Sample photos showing the Fos expression in the L5 SDH of SNI rats. **(i)** Summarized results showing the number of Fos-immunopositive neurons in laminas I-V of SDH of sham rats. */** indicates p < 0.05/0.01 in comparison with sham group (Lamina I: sham+ACC stimuli., p = 0.01; sham+lido.+ACC stimuli., p = 0.01. Lamina II: sham+ACC stimuli., p = 0.005; sham+lido.+ACC stimuli., p = 0.02. Lamina IV: Left, sham+lido., p = 0.04; sham+lido.+ACC stimuli., p = 0.04; Right, sham+lido., p = 0.05; sham+lido.+ACC stimuli., p = 0.04. Lamina V: Left, sham+lido., p = 0.02; sham+lido.+ACC stimuli., p = 0.05; Right, sham+lido., p = 0.007; sham+lido.+ACC stimuli., p = 0.04). ϕ indicates p < 0.05 in comparison with sham+lido. group (lamina I: p = 0.04; Lamina II: p = 0.03). One-Way RM ANOVA. **(j)** Summarized results showing the number of Fos-immunopositive neurons in laminas I-V of SDH of SNI rats. #, p> 0.05 compared with SNI group.

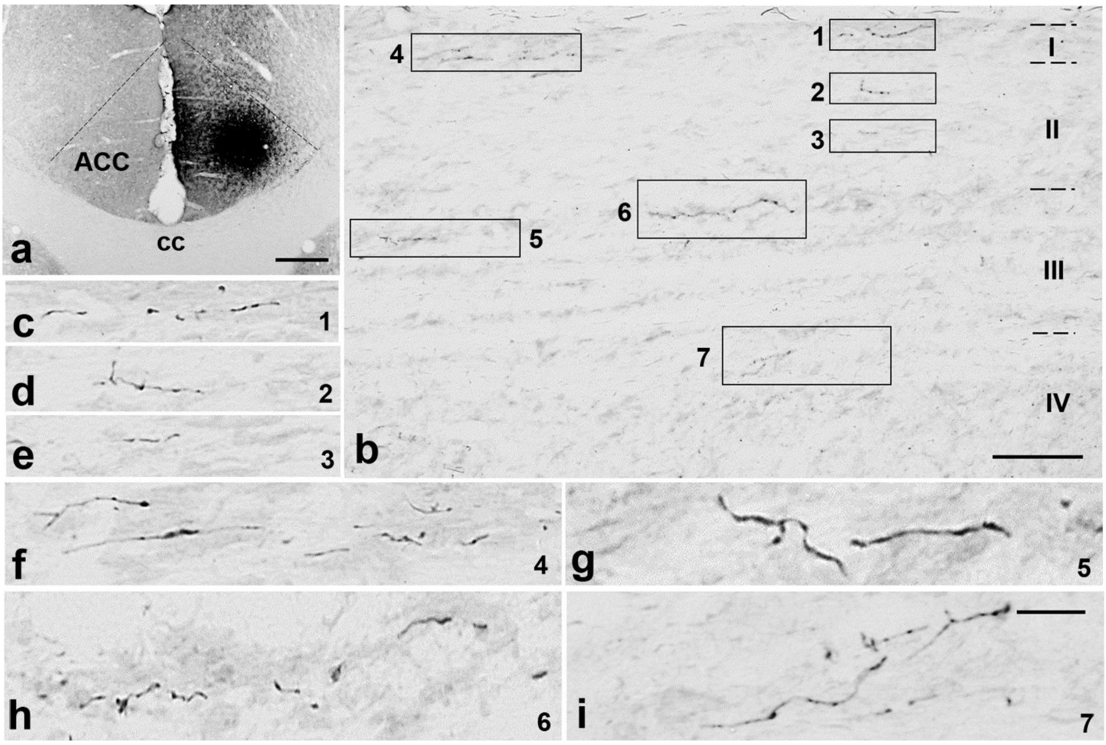

Supplementary Figure 3 | Distribution of PHA-L anterograde labeled fibers and terminals in the spinal dorsal horn projected from the ACC. (a-b) One sample figure from a sagittal slice **(b)** showing that Pha-L labeled fibers and terminals were distributed in the laminae I-III of the spinal cord after PHA-L injection into the contralateral side of the ACC **(a)**. **(c-i)** The rectangled areas **(**1–7**)** in **(b)** were augmented respectively. Bars equal to 1000 μm in **(a)**, 200 μm in (**b)** and 25 μm in **(c-i).**

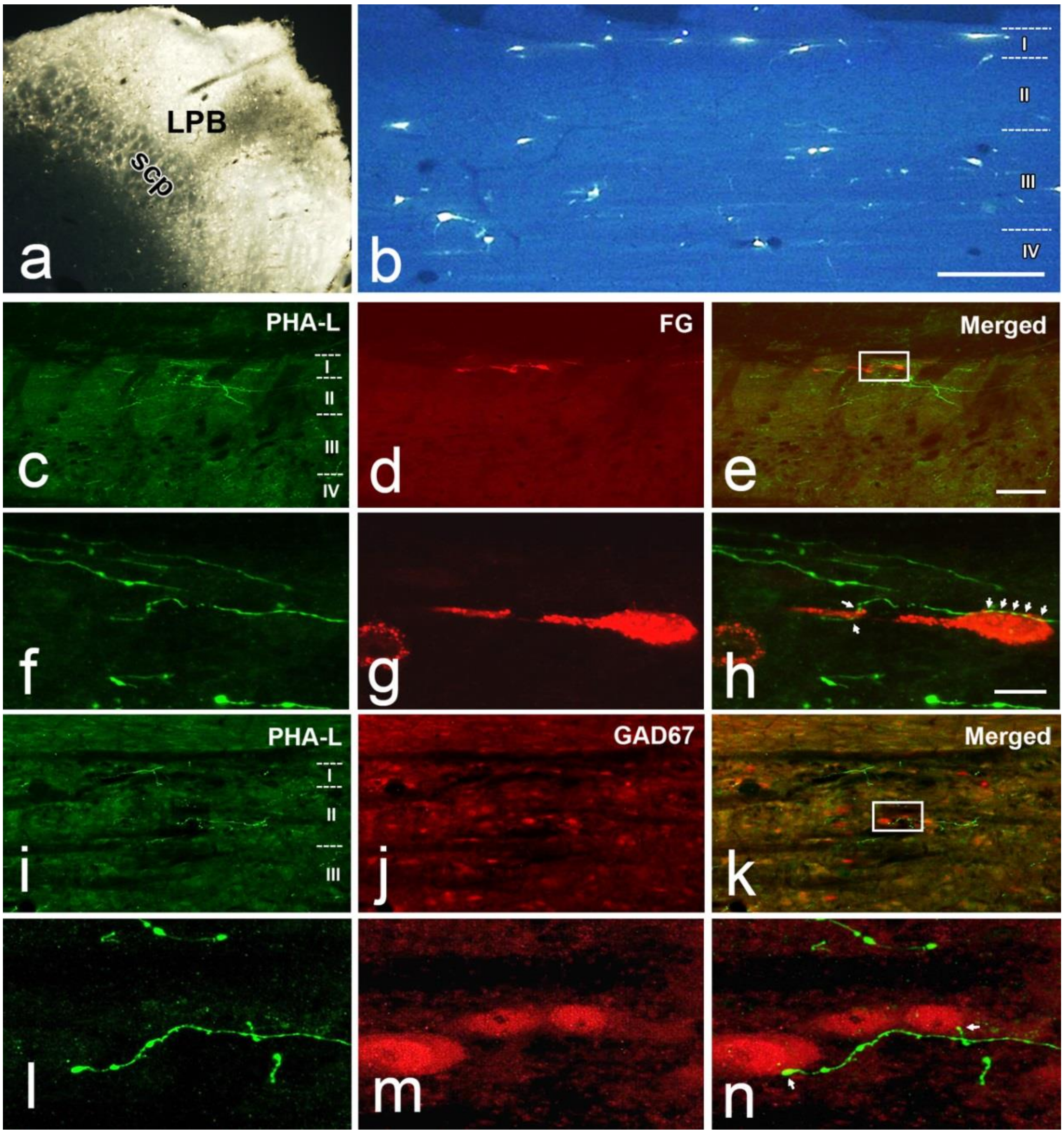

Supplementary Figure 4 | ACC-spinal cord projecting fibers and terminals made close connection with SDH neurons. (a) Fluorescent photomicrograph showing FG injection site in the PBN. **(b)** The general distributing patterns of FG retrogradely-labeled neurons in the sagittal sections of the SDH contralateral to the FG injection site. **(c-h)** Representative fluorescence photomicrographs displaying the close contacts between PHA-L-labeled fibers and terminals and FG-labeled neurons. The images from **(c-d)** are merged in **(e)**. The rectangular areas in **(e)** was enlarged and displayed in **(f-h)**. **(i-n)** Close appositions between PHA-L-labeled fibers and terminals and GAD67-IR neurons of the SDH. The images from **(i-j)** are merged in **(k)**. The rectangular area in **(k)** was enlarged in **(l-n)**. White arrows indicate the close contacts between the PHA-L-labeled terminals and fibers (green) and FG-labeled neuron (red) **(h)** or GAD67-IR neurons (red) **(n)**. Bars equal to 200 μm in **(a-b)**, 100 μm in **(c-e, i-k)** or 10 μm in **(f-h, l-n)**.
