# Supplementary material for: Top-down descending facilitation of spinal sensory excitatory transmission from the anterior cingulate cortex
Source: Nat Commun. 2018 May 14;9:1886. doi: 10.1038/s41467-018-04309-2 (PMC5951839; doi:10.1038/s41467-018-04309-2)
Supplement: Supplementary file 3 — Description of Additional Supplementary Files [file 41467_2018_4309_MOESM3_ESM.pdf]

Description of Additional Supplementary Files

File Name: Supplementary Movie 1
Description: **Pinching induced Ca^{2+} responses in SDH GCaMP6-expressing neurons before and after ACC stimulation.** Left, Pinching induced Ca^{2+} responses in basal condition. Right, Pinching induced Ca^{2+} responses after ACC stimulation.

File Name: Supplementary Movie 2
Description: **Brushing induced Ca^{2+} responses in SDH GCaMP6-expressing neurons before and after ACC stimulation.** Left, Brushing induced Ca^{2+} responses in basal condition. Right, Brushing induced Ca^{2+} responses after ACC stimulation.
